# Anthracycline-induced cardiotoxicity: mechanisms, monitoring, and prevention

**DOI:** 10.3389/fcvm.2023.1242596

**Published:** 2023-12-19

**Authors:** Yun Qiu, Piao Jiang, Yingmei Huang

**Affiliations:** ^1^ Department of Cardiology, Jiangxi Provincial People’s Hospital, The First Affiliated Hospital of Nanchang Medical College, Nanchang, China; ^2^ Department of Oncology, Jiangxi Provincial People’s Hospital, The First Affiliated Hospital of Nanchang Medical College, Nanchang, China; ^3^ The First Clinical Medical College, Nanchang University, Nanchang, China

**Keywords:** anthracycline, cardiotoxicity, mechanisms, monitoring, prevention

## Abstract

Anthracyclines are the most fundamental and important treatment of several cancers especially for lymphoma and breast cancer. However, their use is limited by a dose-dependent cardiotoxicity which may emerge early at the initiation of anthracycline administration or several years after termination of the therapy. A full comprehending of the mechanisms of anthracycline-induced cardiotoxicity, which has not been achieved and is currently under the efforts, is critical to the advance of developing effective methods to protect against the cardiotoxicity, as well as to early detect and treat it. Therefore, we review the recent progress of the mechanism underlying anthracycline-induced cardiotoxicity, as well as approaches to monitor and prevent this issue.

## 1. Introduction

Anthracyclines are one of the most commonly used chemotherapies. Since found in the 1950s, anthracyclines have become the footing stone of chemotherapies in various cancers, especially in breast cancer and lymphoma. Even though anthracyclines improve long-term survival from cancers, it comes accompanied by increased incidence of cardiovascular complications. Since 1971, a dose-dependent cardiotoxicity induced by anthracyclines has been found (1). Indeed, anthracyclines are the major reason of chemotherapy-mediated cardiotoxicity. Clinically, cardiotoxicity can be asymptomatic or manifest diverse symptoms like arrhythmia, declined systolic function, and myocarditis, which can arise promptly upon anthracycline treatment, or arise after several years. In some cases, cardiotoxicity can even impact the option of cancer therapies (2, 3). When the declined heart functionalities or other evident symptoms arise, patients may be limited to choose ineffective therapies, or the theraputic strategy can even be suspended.

Anthracycline-induced cardiotoxicity refers to a series of cardiovascular complications with no definite definition, as a result, its incidence is different on the basis of its definition. Congestive heart failure (CHF) is found in 2%–4%, subclinical structural alterations in about 10%, arrhythmia in over 12%, and cardiac biomarker elevation in 30%–35% of patients (4). A prospective trial including 2,625 patients with an average follow-up of 5.2 years showed a 9% rate of occurrence of anthracycline-mediated cardiotoxicity (5). In this study, anthracycline-mediated cardiotoxicity was defined as left ventricular ejection fraction (LVEF) <50% and a reduction of >10 percentage. Besides, it's reported that childhood cancer survivors showed a 15-fold augmented risk of CHF as well as a 7-fold augmented risk of premature death (6). It's well validated that anthracycline-induced cardiotoxicity is dose-dependent. Bernstein et al. (7) showed that left ventricular (LV) dysfunction which represented a decrease in ejection fraction of >10% lower than normal, emerged in 10%, 16%, 32%, and 65% at total doxorubicin doses of 250, 300, 400, and 550 mg/m^2^, separately. Therefore, even at the lowest dose, anthracyclines are capable of causing remarkable LV dysfunction.

Unfortunately, there are no definite guidelines for screening, monitoring, preventing or treating cardiotoxicity in patients receiving anthracycline administration. Also, comprehending the mechanisms of anthracycline-induced cardiotoxicity is critical to the advance of effective methods to protect against the cardiotoxicity, as well as to early detect and treat it. Therefore, we review the recent progress of the mechanism underlying anthracycline-induced cardiotoxicity, as well as approaches to monitor and prevent this issue.

## 2. Anthracycline-induced cardiotoxicity

It was not until 1967 that the cardiotoxic effects of anthracyclines were identified, following observations by Tan et al. linking them to CHF (8). The cardiotoxicity associated with anthracyclines originally manifest as subclinical cardiomyocyte injuries and gradually lead to early asymptomatic decline of LVEF and eventually symptomatic, and usually intractable, heart failure (HF). Currently, no formal definition of anthracycline-induced cardiotoxicity is achieved. Usually, cardiotoxicity refers to existence of symptoms of HF or asymptomatic reduction of LVEF ≥10% to eventual LVEF <55%.

Generally, anthracycline-induced cardiotoxicity may be categorized into three types: acute cardiotoxicity, early-onset chronic cardiotoxicity, and late-onset chronic cardiotoxicity (9, 10). Acute cardiotoxicity, which occurs in only 1% of patients and typically manifests within the first few days following anthracycline administration, is characterized by supraventricular arrhythmia, transient LV dysfunction, and electrocardiographic alterations (11). Although acute cardiotoxicity manifestations are generally reversible, they are possible to progress into chronic cardiotoxicity. Unfortunately, there are currently no valid approaches for predicting the cardiotoxicity progression.

Early-onset chronic cardiotoxicity, which is the most common type of anthracycline-induced cardiotoxicity and typically manifests within the first year subsequent to termination of anthracycline therapies, is featured by LV dysfunction, reduced LVEF as well as symptomatic HF in about 5% of patients (12).

As for late-onset chronic cardiotoxicity, which is typically irreversible, it may occur a few years or decades after anthracycline therapies (13). This toxicity has been considered as an advanced outcome of early-onset cardiotoxicity with superimposed cardiac injury, primarily manifesting as cardiomyopathy, which features cytoplasmic vacuolization of cardiomyocytes due to sarcoplasmic and mitochondrial swelling, organelle destruction, cardiomyocyte death, as well as muscle fiber disorder (14). These alterations result in an irreversible decrease of LVEF in approximately 26% of patients and symptomatic HF in over 7% of patients (9, 15).

Interestingly, Cardinale et al. questioned the current classification of anthracycline-induced chronic cardiotoxicity which regards early-onset and late-onset chronic cardiotoxicity as two different entities on the basis of retrospective studies in which LVEF decrease was tested either after HF development, or on random assessment in pediatric cancer patients presumed to have no other cardiac complications. Nevertheless, Cardinale et al. demonstrated that HF may be preceded by asymptomatic LVEF reduction featured by early onset with a slow and gradual deterioration that may sustain months or years after the termination of anthracycline treatment (5).

## 3. Mechanism of anthracyclines-induced cardiotoxicity

**Figure 1:**
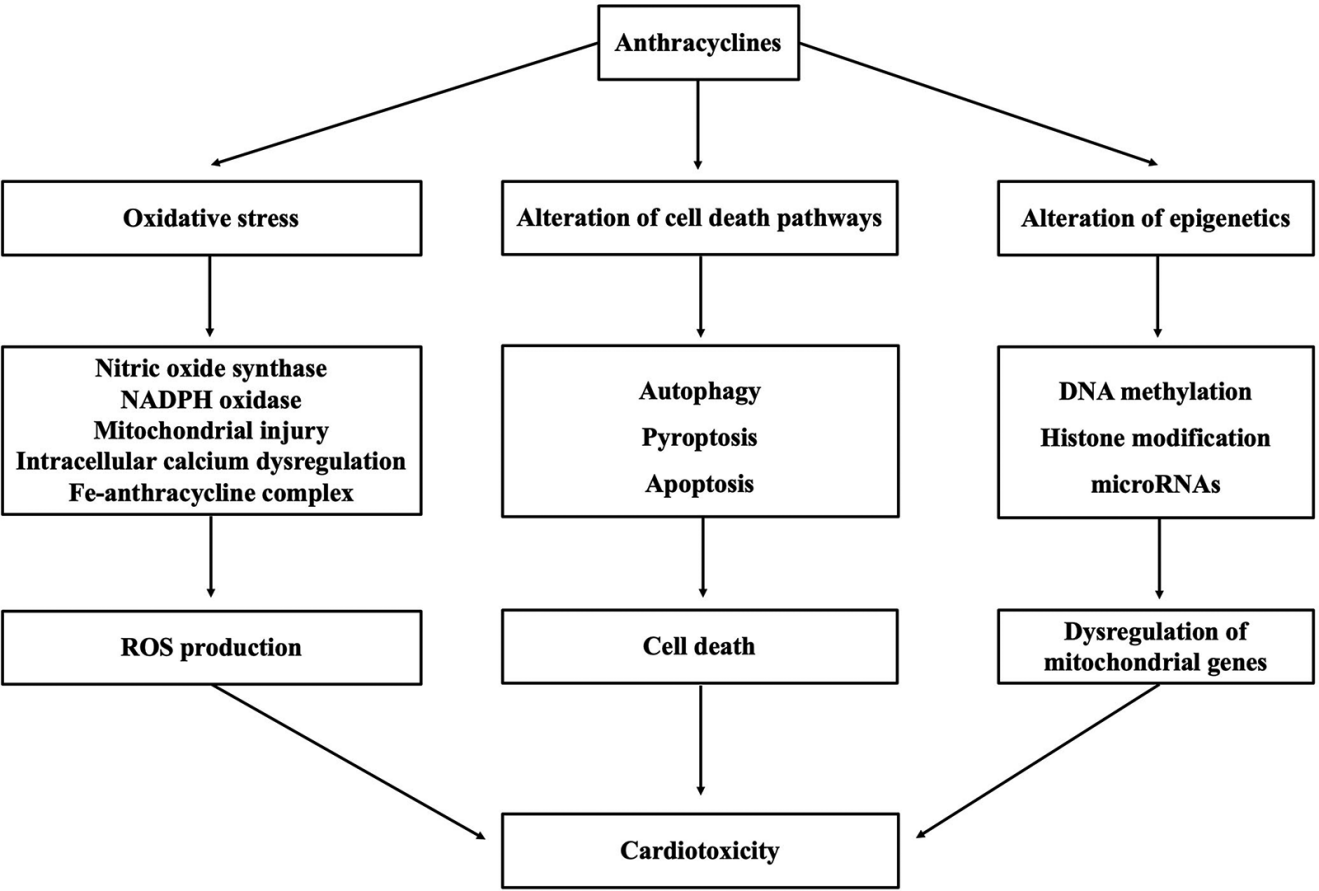
Summary of mechanisms underlying anthracycline-induced cardiotoxicity.

Although the mechanism underlying cardiotoxicity caused by anthracyclines is not well comprehended, proposed mechanisms include oxidative stress triggered by various molecular mechanisms, alteration of cell death pathways, and alteration of epigenetics (as shown in Figure 1).

### 3.1. Oxidative stress

Oxidative stress, caused by the imbalance between reactive oxygen species (ROS) and antioxidants, is the leading concern of anthracycline-induced cardiac muscle cell injuries (16). A certain levels of oxidant species are essential for normal signal transduction process; however, excessive oxidant species have been proven to be related to loads of pathological conditions. As reported, an evident growth of oxidative stress is observed when the total dose of doxorubicin surpasses 500 mg/m^2^ (17). The molecular mechanisms triggering oxidative stress and its damaging effects on heart tissue are concluded as below.

#### 3.1.1. Effect of nitric oxide synthase on ROS production

Nitric oxide (NO), generated by catalyzation of nitric oxide synthase (NOS), is a primary reason of anthracycline-mediated oxidative stress. And NO levels have been proved elevated in cardiotoxicity associated with anthracycline administration. There are three types of NOS, namely endothelial NOS (eNOS), inducible NOS (iNOS) and neuronal NOS (nNOS), with the first two playing major roles in cardiac NO synthesis. And an upregulated level of iNOS and eNOS has been observed in cardiomyocytes treated with anthracyclines.

Anthracyclines, by binding to the eNOS reductase, boost semiquinone radical generation which reduces the free oxygen into the superoxide (O_2_^–^) free radical. An enzymatic single-electron reduction reaction is accomplished with the assistance of flavoenzymes including NADPH-cytochrome P450 reductase and mitochondrial NADH dehydrogenase (18). An imbalance between superoxide free radicals and NO levels is produced after anthracyclines bind to the eNOS reductase. ROS increase the formation of oxidants and afterwards compel the uncoupling of eNOS into monomers, thus promoting the generation of increased superoxide anions as well as declined NO which finally results in cardiotoxicity (19). Neilan et al. verified the important effect of eNOS on doxorubicin-mediated oxidative stress by eNOS knockout mice which exhibited low-level ROS and preserved myocardial function after treated with doxorubicin. Furthermore, an increased deleterious action of anthracyclines on the heart tissue was observed in the eNOS-overexpressed cardiomyocyte (20). Another study suggested that antisense eNOS mRNA decreased the activity of caspase-3, revealing its cardioprotective action on doxorubicin-mediated cardiotoxicity. Similarly, increased anthracycline-mediated ROS generation in the cardiomyocyte was observed in transgenic eNOS mice (21).

In addition to eNOS, anthracycline treatment also promotes the iNOS mRNA and protein levels and contributes to the production of nitrotyrosine (NT) with enhanced mitochondrial superoxide level in heart tissue (22). The generation of superoxide and NT depends on the peroxynitrite level related to the production of powerful oxidants such as nitrogen dioxide and carbonates radical which leads to myocardial cell apoptosis, declined cardiac contractility, and declined glutathione peroxidase activity after anthracycline treatment (23).

#### 3.1.2. Effect of NADPH oxidase on ROS production

NADPH oxidase (Nox), a significant source of cardiac ROS generation, is a multi-component enzyme complex composed of a catalytic core (Nox2/p22phox) and cytosolic regulatory subunits (24). Seven isoforms of NOX have been identified, and NOX2 isoform is the principal one involved in anthracycline-mediated cardiotoxicity and Nox-induced ROS. Research showed that NADPH-dependent superoxide formation was significantly declined in Nox2^−/−^ mouse models compared with that in wild type (WT) mice. Besides, Nox2 mRNA levels were raised after anthracycline treatment in WT hearts but were undetectable in Nox2^−/−^ hearts (25). Noxs, which are usually activated by stimuli like AngII, cyclic stretch and TNF-α which are crucial in cardiac remodeling, can also regulate other ROS sources. For instance, Nox-induced ROS enable the stimulation of oxidative degradation of tetrahydrobiopterin, resulting in NOS uncoupling and superoxide generation (26). Nox is related to anthracycline-induced cardiotoxicity as a significant source of anthracycline-dependent ROS. Like NOS, Nox oxidizes the quinone structure of anthracyclines into a semiquinone radical with NADPH offering electrons. Then semiquinone radicals react with oxygen to produce H_2_O_2_ and superoxide, which can impair DNA. On the other hand, anthracyclines stimulate the activation of Nox, leading to ROS production (27).

#### 3.1.3. Effect of mitochondrial injury on ROS production

Anthracyclines primarily target the cardiomyocytes' mitochondria, thus resulting in impaired cardiomyocyte. Anthracyclines have a potent affinity with cardiolipin which is a significant composition of the inner mitochondrial membrane, thus generating an irreversible compound through electric force to promote cardiotoxicity (28). After the anthracycline treatment, it binds to cardiolipin, leading to the impaired cardiolipin functioning and the production of superoxide radical (29). Emerging evidence demonstrates that anthracyclines are capable of quickly and directly intercalating into mitochondrial DNA (mtDNA) *in vivo*, leading to nucleoid aggregation and mtDNA reduction (30). Once anthracyclines intercalate to mtDNA, the mitochondrial oxidative respiratory electron transport chain will be arrested as a result of oxidation of mtDNA proteins like cytochrome C oxidase and NADH dehydrogenase, thus resulting in ROS upregulation, energy decline as well as cell apoptosis. Furthermore, a shift in the metabolism of cardiomyocyte mitochondria after doxorubicin administration have been observed that doxorubicin decreases the oxidation of long-chain-fatty acid but enhances glucose metabolism, which probably cause HF or abnormal cardiac relaxation and contraction (31, 32). In addition, anthracyclines hinder the expression of GATA-4 gene in the mitochondria, thus inhibiting mitochondrial synthesis and metabolism and enhancing apoptosis (33). Recent metabolomic studies highlight the possibility that cardiac metabolic alterations may critically contribute to the development of doxorubicin cardiotoxicity (34) and the metabolic alterations reduced ROS production and improved mitochondrial dysfunction in cardiomyocytes (35).

#### 3.1.4. Intracellular calcium dysregulation

Anthracycline cardiotoxicity is related to impaired calcium homeostasis caused by doxorubicin-induced damage of cell membrane permeability, which results in intracellular calcium upregulation, ROS formation, and resultant apoptosis in cardiomyocytes, whereas Ca^2+^ chelators significantly attenuate the anthracycline-induced ROS formation and cardiomyocyte injuries (36). Moreover, anthracycline-induced cardiomyopathy is concerned with changes of the calcium exchange regulating gene, which has an effect on ryanodine receptor, calcium storage gene, Sarco/endoplasmic reticulum ATPase (SERCA2a), and phospholamban, eventually leading to damage of cardiac systolic and diastolic abilities. Notably, research shows that the removement of doxorubicin metabolite is rather intractable compared with doxorubicin, which causes the accumulation of doxorubicin metabolite on cardiomyocytes and leads to remarkable calcium dysregulation, damaging heart tissue (37).

In addition, calpains, calcium-dependent proteases, play an important role in intracellular calcium dysregulation. A rise of calcium level in the sarcoplasmic reticulum of cardiomyocytes has been found under oxidative stress and results in leakage of calcium ions which stimulate the calpains. These calpains cleave the caspase-12 and induce cardiomyocyte apoptosis. Anthracycline-induced cardiotoxicity is also associated with the devastation of cardiac muscle proteins, which emerges due to the activation of calpains, which degrade the titin, a major composition of cardiac sarcomere, thus causing cardiotoxicity (38).

#### 3.1.5. Fe-anthracycline complex

Anthracyclines can interact with iron to form compounds which cycle between Fe(II) and Fe(III), leading to remarkable oxidative stress and mitochondrial dysfunction (39). Doxorubicinol, a derivative of doxorubicin, sequesters iron from the iron-sulfur cluster of iron regulatory protein 1 (IRP1), resulting in its inactivation. Inactive IRP1 impairs cellular iron homeostasis via mounting expression of the cellular iron importer protein transferrin receptor but weakening expression of the cellular iron storage protein ferritin, causing enhanced cellular free iron uptake and diminished sequestration, separately (40). And augmented free iron in the myocardial cells are catalysts for intracellular ROS generation. Deficiency of human hereditary hemochromatosis (HFE) gene enhanced mitochondrial ROS generation and evidently exacerbated HF caused by doxorubicin in mice with iron overload in contrast to control group (41). Furthermore, Mfrn-2 is a transporter participated in iron access to the mitochondria. Mfrn-2 level is associated with the absorption in iron mitochondria. And Mfrn-2 deletion destroys the iron homeostasis in the mitochondria (42), indicating the critical effect of free iron on anthracycline-mediated cardiotoxicity and that decreasing iron levels can be a potential way to avoid anthracycline-mediated cardiotoxicity.

### 3.2. Alteration of cell death pathways

#### 3.2.1. Autophagy

Autophagy is a homeostatic course through which cellular components are degraded and recycled in normal and stress conditions. Indeed, anthracycline can stimulate autophagy, however, it is the deregulation of autophagy that results in extravagant myocardial cell death. It is debatable whether anthracycline enhances or disturbs autophagy in cardiac tissue. In the initiation stage, some evidence demonstrates that anthracycline increases AMPK (43–45), while others demonstrates no alterations (46, 47) or a reduction in AMPK activation (48, 49). Fortunately, recent studies have facilitated to settle this discrepancy via assuming that anthracycline firstly stimulates autophagy but afterwards intercepts it (50, 51) leading to the accumulation of undegraded autophagosomes and autolysosomes that worsen the destruction in myocardial cells. Indeed, a low dose of anthracyclines causes the elevated level of LC3-II, p62, and Beclin1 protein, suggesting stimulation of autophagy. Nevertheless, when evaluating downstream circumstances of autophagy, anthracyclines destroy the autophagic flux and restrict lysosomal acidification in myocardial cells. The deregulation of autophagy causes the accumulation of undegraded autolysosomes, which induces ROS generation and anthracycline-mediated cardiotoxicity (52). Mice with Beclin 1 haplo deficiency were observed declined autophagy initiation ability and thus a decreased amount of undegraded autolysosomes compared with the wildtype mice after anthracycline administration, therefore resulting in a decline of ROS generation and eventual anthracycline-induced cardiotoxicity. Conversely, enhancing the activity of autophagy via upregulating Beclin 1 worsens the cardiotoxicity (52).

#### 3.2.2. Pyroptosis

Pyroptosis has been proven an important effect on the pathogenesis of cardiovascular diseases. Anthracycline-induced pyroptosis proceeds through the increase of Terminal Differentiation-Induced Non-Coding RNA (TINCR), which recruits IGF2BP and enhances the expression of NLRP3 resulting in activation of caspase-1, the cleavage of GMDSD-N, as well as the release of IL-1β, IL-18. The inhibition of NLRP3 with MCC950 prevented the cardiomyocytes from anthracycline-induced cell death (53). Sun et al. showed that Sirtuin 1 activation inhibited NLRP3 and protected cardiomyocytes from anthracycline-induced pyroptosis (54). Protection from anthracycline-induced cardiotoxicity has also been achieved via the inhibition of the NLRP3/caspase 1 signaling with embryonic stem cell-derived exosomes, upregulation of heat shock protein 22, and pharmacologic inhibition of NLRP3 (55). In addition, anthracycline can trigger pyroptosis by the stimulation of Bnip3 in the mitochondria. Anthracycline enhances the expression of Bnip3 which stimulates caspase 3 and results in GSDME-dependent pyroptosis. Destruction of GSDME and silencing of Bnip3 avoids cardiomyocyte death induced by anthracyclines *in vitro* (56).

#### 3.2.3. Apoptosis

Apoptosis is the most researched programmed cell death pathway in anthracycline-induced cardiotoxicity. Anthracycline administration results in excess oxidative stress (lipid peroxidates, doxorubicin's reduced semiquinone moiety, charged doxorubicin iron complex, respiratory chain failure, peroxynitrites, etc.) and mitochondrial damage which triggers cell death pathways including apoptosis.

Anthracycline can trigger the intrinsic apoptosis pathway through Bax/Bak stimulation and translocation from the cytosol to the outer membrane of mitochondria causing the mitochondrial outer membrane permeabilization, enabling the diffusion of certain proteins to the cytoplasm like pro-apoptosis factor cytochrome c (57). Various mechanisms participate in anthracycline-induced intrinsic apoptosis activation, involving overexpression of p53 that results in Bax overexpression, decline of GATA4 that reduces antiapoptotic Bcl-XL level, activation of JNK and MAPK, and inactivation of PI-3K/Akt pathway (58).

Anthracycline also trigger the extrinsic apoptosis pathway in cardiomyocytes. Death ligands, such as FasL and TNFα, bind to their receptors and stimulate the recruitment of cytosolic proteins Fas-associated via death domain (FADD) and TNFR-associated death domain (TRADD) (59). FADD and TRADD recruit caspase 8, and activated caspase 8 is capable of activating caspase 3 which causes apoptosis. Mechanisms related to anthracycline-induced extrinsic apoptosis activation include activation of nuclear factor-activated T cell-4 (NFAT4) and NF-κB, resulting in the upregulation of Fas/FasL and p53; and, downregulation of FLIP, a FLICE/caspase-8 inhibition protein, which causes Fas-mediated cell death (58).

Recent data indicates that circulating death ligands may serve as serum biomarkers, therefore comprehending the extrinsic apoptosis pathway may be helpful for developing treatment strategies. McSweeney et al. explored doxorubicin-caused transcriptomic alterations by RNA-sequencing (RNAseq) and a cellular model consisting of human induced pluripotent stem cell-derived cardiomyocytes (hiPSC-CMs). Results showed p53 protein was a critical regulator of transcriptomic alterations associated with doxorubicin. Furthermore, overexpressed death receptor and upregulated extrinsic apoptotic pathway were demonstrated obvious correlation with doxorubicin-mediated cardiotoxicity (60). Zhao et al. studied doxorubicin-mediated cardiotoxicity in human induced pluripotent stem cells-derived cardiomyocytes (iPS-CMs). They discovered that doxorubicin remarkably upregulated the expression of death receptors (TNFR1, Fas, DR4 and DR5) in iPS-derived cardiomyocytes at both protein and mRNA levels. The resulting iPS-CMs cells underwent spontaneous apoptosis which was further enhanced by physiologically relevant death ligands including TNF-related apoptosis inducing ligand (TRAIL). Furthermore, TRAIL potentiated doxorubicin-induced decrease in beating rate and amplitude of iPS-derived cardiomyocytes. These data demonstrate that the induction of death receptors in cardiomyocytes is likely a critical mechanism by which doxorubicin causes cardiotoxicity (61).

### 3.3. Alteration of epigenetics

#### 3.3.1. DNA methylation

Anthracycline downregulates the activity of DNA methyltransferase 1 (DNMT1), thus hindering the DNA methylation procedure. This hypomethylation causes disordered mitochondrial gene expression including peroxisome proliferator-activated receptor-gamma coactivator-1-alpha (PGC-1α), nuclear respiratory factor 1 (NRF-1) and mitochondrial transcription factor A (TFAM) unit in the rat cardiac tissue upon doxorubicin treatment (62), all of which can eventually cause cardiomyocyte dysfunction.

Ferreira et al., used mouse cardiomyocyte H9c2 cells to investigate the role of doxorubicin and found a reduction in glycolysis and basal respiration, as well as disordered mitochondrial DNA transcription after doxorubicin treatment (63). Notably, decrease of DNMT1, along with a global DNA hypomethylation, was found as well. These findings were consistent with earlier research in which global reduction of DNA methylation and tangled mitochondrial gene expression were found in the cardiac tissue of rats with doxorubicin treatment (62). Interestingly, their work showed that pre-exposure of doxorubicin could endow resistance to the following administration of doxorubicin in H9c2 cells, perhaps resulting from mitochondrial adaptation (63).

#### 3.3.2. Histone modifications

Apart from DNA methylation, histone modifications play a role in anthracycline-mediated cardiotoxicity as well. Histone modifications can produce synergistic or antagonistic interactions with chromatin-associated protein, leading to dynamic switching between transcriptionally active and silent status. For instance, histone deacetylase, HDAC6, was proven overexpressed in doxorubicin-treated primary rat cardiomyocytes and mice, leading to deacetylation of α-tubulin. Genetic or pharmacological inhibition of HDAC6 in mice exhibited a cardioprotective effect against doxorubicin by restoring autophagic flux (64). Moreover, Hanf et al., using H9c2 cardiomyocytes, also confirmed that disordered expression of some histone modifiers was related to decrease of global acetylation of histone H3. The expression of histone deacetylases (SIRT1 and HDAC2) was impacted by doxorubicin administration. As for HDAC2, treatment with low-dose doxorubicin reduced its expression, however, no obvious alterations were observed in high dose treatment in contrast to control (65).

#### 3.3.3. microRNAs (miRNAs)

Another epigenetic modification involved in anthracycline-induced cardiotoxicity is the regulation of miRNAs. Abnormal levels of some miRNAs have been demonstrated associated with anthracycline-induced cardiotoxicity. For instance, overexpression of miR-15 was found in doxorubicin-mediated apoptotic H9c2 cells (66). This phenomenon was maybe owing to the inhibition of Bmpr1a, a target of miR-15 and BMP receptor, reported to participate in myocardial contractility. As a result, activation of BMP signaling with Bmpr1a agonist is capable of mitigating doxorubicin-induced cardiotoxicity in H9c2 cells. Besides, overexpression of miR-23a, miR-34a, miR-140, miR-146a, and miR-532 were found *in vitro* and/or *in vivo* models of anthracycline-caused cardiotoxicity (67). Notably, overexpression of miR-34a, a famous tumor suppressive miRNA, could epigenetically inhibit SIRT1, thereby somewhat elucidating the decrease of the HDAC induced by doxorubicin, in the previously mentioned research (65). Furthermore, adenovirus-mediated upregulation of miR212/132 demonstrated protection against doxorubicin-caused cardiotoxicity in mice (68). Mechanism underlying this may be direct targeting of Fitm2, a transmembrane protein associated with fat storage, via miR-232/132.

## 4. Monitoring

In 2014, Cancer Therapeutics-Related Cardiac Dysfunction (CTRCD) was formally defined by the American Society of Echocardiography (ASE) and the European Association of Cardiovascular Imaging (EACVI) as a reduction of the LVEF by 10 percentage to below 53% through echocardiography (69, 70). CTRCD can be classified into four groups: “Reversible” when the LVEF recovers to within 5% of the baseline; “Partially reversible” when the LVEF increases by over 10% from the lowest yet still reserves 5% lower than the baseline; “Irreversible” when the LVEF increases by less than 10% from the lowest yet reserves over 5% lower than the baseline; “Indeterminate” when the patient cannot be reevaluated. As a result, the early detection of anthracycline-induced cardiotoxicity in patients prior to the occurrence of clinical manifestations exerts a crucial impact on the prognosis, making cardiac function monitoring before, during, and after anthracycline treatment significantly essential. Cardiotoxicity monitoring modalities generally involve echocardiography, electrocardiogram (ECG), cardiac magnetic resonance (CMR), nuclear cardiac imaging (MUGA), serum biomarkers, etc. Each method has its own superiorities and inferiorities as shown in Table 1.

**Table 1:** Summary of methods for monitoring cardiotoxicity.

Methods	Superiority	Inferiority	Other
Echocardiography	Detect LVEF, LV wall stress, LV mass, LV thickness-to-size ratio, diastolic function, GLS, etc.	Unable to identify subclinical cardiomyocyte structural alterations	As routine examination
ECG	Convenient and affordable	Insufficient specificity	As routine examination
CMR	Offer all-round information about structure and function	High cost and strict compliance requirements	Preferred
	Multiparameter and multisequence		
	Excellent repeatability		
MUGA scan	Outweighs standard two-dimensional echocardiography in accuracy and repeatability of LVEF detection; able to map molecular processes perturbed in anthracycline-induced cardiotoxicity through radioactively labeled probes	Quick development of other imaging techniques has reduced the application of MUGA due to commensurate outcomes derived from CMR and 3D echocardiography	Application on demand
Biomarkers	Cardiac troponin, NPs, BNP and NTroBNP are sensitive biomarkers of cardiomyocyte injury	Current detection techniques are not sensitive enough	Concomitant use with imaging modalities

### 4.1. Echocardiography

Echocardiography is the most commonly applied monitoring technique for patients undergoing anthracycline therapy (71). Clinically, the most common parameter for the identification and monitoring of anthracycline-induced cardiotoxicity is LVEF (6). Furthermore, the calculation of two-dimensional echocardiography (2DE) LV volume and LVEF has been proposed by the American Society of Echocardiography (ASE) and the European Association of Echocardiography (EAE) as an upgradation of the Simpson technique. Notably, in comparison to the LVEF alone, LVEF combined with an evaluation of the wall motion score index was proven a more sensitive and effective parameter for monitoring of anthracycline-induced cardiotoxicity (72). When 2 or more out of 16 myocardial segments are not capable of sufficiently exhibiting the endocardial border, echocardiography contrast agents are suggested, which can improve the accuracy and repeatability of echocardiography (73).

Recently, myocardial strain abnormalities have been demonstrated earlier than cardiac dysfunction in anthracycline-induced cardiotoxicity (74). And the ESC guidelines suggest the best parameter for strain is the global longitudinal strain (GLS) which displays prognostic information such as postmyocardial infarction, aortic stenosis, HF and myocarditis (75–77). For instance, 1% improvement in GLS of patients with HF is linked to a 5% declined risk of death (75). Thavendiranathan et al. demonstrated that GLS-guided cardioprotective treatment can significantly defer the impairment of cardiac function compared with LVEF-guided group through comparing the reduction of LVEF between the two groups (78). Furthermore, a 15% decrease in GLS compared to baseline value is regarded an early signal of cardiac toxicity and can forecast subclinical LV systolic dysfunction, but a decrease of <8% excludes the diagnosis (79). Notably, strain packages among distinct manufacturers may be diverse, and there is no definite and standard value for determining the abnormal strain. In view of this condition, it is suggested to carry out follow-up investigations utilizing the same manufacturer to realize reproducibility.

### 4.2. Electrocardiogram

Electrocardiogram (ECG), a routine evaluation technique, can show the electrophysiological activity of the heart before, during and after anthracycline administration. Cardiovascular toxicity has various manifestations on ECG, such as sinus tachycardia, QT prolongation, ST-T segment alterations, heart block, etc. (80), among which QT prolongation is of great significance. Andreu et al. summarized the incidence, diagnosis, and outcomes of QT prolongation related to antitumor drugs and concluded that the weighted adjusted incidence of QT prolongation was 0%–22% of patients treated with anthracyclines (81). Ventricular premature contractions are the most common class of arrhythmia in patients receiving anthracyclines and ventricular tachycardia emerges in about 73.9% of patients (82). Mulrooney et al. investigated 2,715 childhood cancer survivors, 10.7% had major ECG alterations and 23.3% showed slight alterations, such as premature contraction, non-specific T wave or ST segment alterations, and low QRS (83). As a result, ECG evaluation is of great significance for patients before, during and after anthracycline administration, particularly for high-risk patients considering that early identification of arrhythmias can improve their prognosis.

### 4.3. Cardiac magnetic resonance (CMR)

Cardiovascular imaging is an important tool in baseline risk assessment and detection of cardiovascular disease in oncology patients receiving cardiotoxic cancer therapies (84). Though echocardiography is a routine examination to detect and monitor the cardiotoxicity caused by anthracyclines, increasing research has confirmed that CMR is an exceedingly reproducible technique in determining LVEF and is the gold standard for the assessment of LV volume and LVEF (85, 86). The advancement of CMR rapid scanning and postprocessing techniques, and the automatic analysis of CMR mapping through machine learning algorithm increases the accuracy and significantly shorten the time for image analysis (87). Jordan et al. investigated whether T1- and T2- weighted measurement of signal intensity was related to declined LVEF in patients receiving anthracyclines and enhanced myocardial contrast echocardiography. Concordant with previous animal studies, the increase in T1-weighted signal intensity was related to myocardial injuries as well as the declined LVEF. The results demonstrated that changes in T1-weighted signal intensity may be an early marker of subclinical myocardial injuries related to the anthracycline treatment (88). Galán-Arriola et al. attempted to detect early cardiotoxicity caused by doxorubicin via serial multiparametric CMR in an animal model. T2 mapping during treatment identified intracardiomyocyte edema generation as the earliest marker of anthracycline-induced cardiotoxicity, in the absence of T1 mapping, ECV, or LV motion defects. The occurrence of these changes at a reversible disease stage indicated the clinical potential of this CMR marker for tailored anthracycline therapy (89).

In addition, CMR exerts a pyramidally conspicuous effect on the identification of cardiotoxicity in multimodal imaging techniques and it may preferably help comprehend the potential mechanisms of anthracycline-induced heart injuries (6). All these results shows that CMR is a very prospective method used to monitor anthracycline-mediated cardiotoxicity. Nevertheless, CMR is limited by high cost, the application of metal devices, serious underlying diseases like advanced renal failure and the like.

Notably, in a lot of research, CMR and echocardiography examinations were generally compared with each other. For, instance, a cohort study of adult survivors from childhood cancers reported approximate average values of LVEF measured by CMR and 3DE respectively, while 2DE values were about 5% higher. Moreover, this research showed that 3DE and 2DE were not the best option for recognizing patients whose LVEFs are lower than a threshold of 50% as determined via CMR (90). In view of this, CMR is the optimal alternative when other methods are useless for determining the LV dysfunction with LVEF lying borderline low.

### 4.4. MUGA scan

Since 1970s, nuclear imaging (also called MUGA) was employed to determine LVEF reduction prior to the occurrence of CHF in patients treated with anthracyclines. MUGA generally outweighs standard r2DE in terms of the accuracy and repeatability of LVEF detection, and an outstanding merit of MUGA is its help in identifying patients with poor echocardiographic windows (71). Researchers explored MUGA scan in anthracycline-induced cardiotoxicity, from pathobiology to the detection of molecular targets. Actually, MUGA scan can map molecular processes perturbed in anthracycline-induced cardiotoxicity via radioactively labeled probes. Molecular targets in nuclear imaging involve metabolic dysfunction, cell death, sympathetic innervation, cardiac fibrosis detection, myocardial perfusion and blood flow measurement. These researchers offered a clinical framework for prospective use of MUGA scan in forecasting and reclassifying cardiotoxicity in cancer patients receiving anthracyclines, as a supplement to echocardiography (91). Notably, the prompt advance of other imaging techniques has reduced the application of MUGA due to commensurate outcomes derived from CMR and 3D echocardiography.

### 4.5. Biomarkers

Biomarkers are extremely crucial for risk evaluation and early determination of cardiotoxicity induced by anthracyclines. The highly sensitive cardiac biomarkers, particularly cardiac troponin (cTn) and natriuretic peptide (NPs), are very promising tools for early identification of subclinical cardiomyocyte alterations and forecasting the following LV dysfunction after the anthracycline administration. Besides, cTn and NPs can also be utilized to instruct the starting of cardioprotection treatment during anthracycline administration and monitor its curative effect (92). Actually, troponin increment is a common accompaniment to anthracycline treatment, and patients whose troponin levels increase are at higher risks of LV dysfunction and resultant LVEF reduction (93). Cardinale et al. reported that 65 out of 204 patients receiving high-dose anthracyclines, exhibited a rise in cTnI (>400 ng/L) and a persistent reduction in LVEF (94). Similarly, a study demonstrated that 15% of the investigated patients suffering from hematologic malignancies exhibited an increment in cTnT (≥0.03 ng/ml) with its summit values detected on day 21, and the increment was related to a LVEF reduction (95).

Notably, though troponin is a highly prospective biomarkers for early detection of anthracycline-induced cardiotoxicity, some obstacles impede its extensive use in clinical practice. First, the optimal timing of troponin evaluation is contradictory as it is indefinite whether a singular measurement of elevated cTn owns adequate predictive value or several measurements may be needed (10). Besides, the cutoff point which maximizes the positive and negative predictive value is still disputed (96).

Furthermore, the level of BNP and NTroBNP during anthracycline administration is related to the decline of LVEF and poor prognosis as well (97). Bhuva et al. investigated a cohort of 333 patients receiving anthracyclines undergoing various cancers and suggested that BNP >100 pg/ml was an indication of long-term HF, yet not a risk factor for all-cause mortality. If BNP cutoff point was 30 ng/L, the negative predictive value of occurrence of HF was 98% (87). The continuous rise of NTroBNP in the early stage upon high-dose anthracyclines is also concerned with the progression of myocardial injuries. A study by De Iuliis et al. demonstrated a remarkable correlation between NTroBNP increased values after anthracyclines administration and prediction of mortality at 1 year in breast cancer patients (97). Additionally, some novel serum biomarkers including myeloperoxidase, high-sensitivity C-reactive protein, SFLT1, placental growth factor, arginine nitric oxide metabolites, glycogen phosphorylase BB and topoisomerase 2β all rose upon anthracycline administration (98, 99), indicating that the combination of multiple markers probably enhance the capacity to identify early cardiac structural abnormalities.

## 5. Prevention

### 5.1. Anthracycline administration

Strategies used to avoid cardiotoxicity caused by anthracycline whereas keeping antitumor efficacy involving extended infusion delivery times, limited lifetime dose and the application of liposomal-encapsulated anthracyclines. Given the linear correlation between peak anthracycline levels and cardiotoxicity, continuous infusion of anthracyclines decreases the risk of cardiotoxicity in contrast to prompt bolus dosing (100, 101). van Dalen et al. investigated various pharmacokinetic infusion durations among 557 patients, and found anthracycline infusion for 6 h or more decreased the risk of HF in contrast to bolus dosing (102). The presumable reason for these phenomena is that the pharmacokinetic determinates of cardiotoxicity are different from the antitumor efficacy. Though the anticancer efficacy is primarily impacted by overall dose of anthracyclines, cardiotoxicity is related to the peak plasma levels (101). However, continuous doxorubicin infusion more than 48 h for childhood leukemia showed no cardioprotection superiority in contrast to bolus infusion (103). Given its dose dependence of anthracycline-induced cardiotoxicity, the total dose is usually confined to 450 mg/m^2^ of doxorubicin, 600 mg/m^2^ of daunorubicin or 900 mg/m^2^ of epirubicin. Analogs of doxorubicin like epirubicin cause comparatively less cardiac injuries, hence possessing higher dose limits. Smith et al. performed a meta-analysis and summarized that epirubicin had lower risks of both clinical and subclinical cardiotoxicity compared with doxorubicin (104). Though cutting down the overall dose of anthracyclines decreases short-term cardiotoxicity, no influence in long-term cardiotoxicity was observed. In children receiving a maximum dose of doxorubicin of 100 mg/m^2^, myocardial structure abnormalities were observed in 30% of patients a few years later (105). Another way to decrease anthracycline-mediated cardiotoxicity is to utilize liposomal encapsulation that enables site-specific delivery of anthracyclines. van Dalen et al. carried out a meta-analysis which showed an evidently declined rate of both clinical HF and clinical and subclinical HF combined in patients receiving liposomal-encapsulated doxorubicin (RR = 0.20, 95% CI 0.05–0.75 and RR = 0.38, 95% CI 0.24–0.59 respectively) compared with conventional doxorubicin (106). Despite their safety and no compromise of antitumor activity, the application of liposomal anthracyclines is confined to ovarian cancer, Kaposi's sarcoma, and multiple myeloma due to their high cost.

Interestingly, research has verified that natural bioactive compounds (NBACs) have a wide range of biological activities related to anticancer outcomes. Combining NBACs with anthracyclines like doxorubicin has been considered to be an ideal candidate that might increase the effectiveness of doxorubicin therapy and decreases its unfavorable adverse consequences like anthracycline-induced cardiotoxicity. Elfadadny et al. (107) showed that NBACs possess a high binding affinity to topoisomerase II-alpha ranged from 2.42 to 7.41 pKi that enhance the anticancer effect when combined to doxorubicin. Also, they demonstrated that NBACs-doxorubicin combination alleviates the doxorubicin-induced cardiotoxicity through reduction of ROS production and increases the activity of antioxidant enzymes, indicating that combination of NBACs and doxorubicin may be a very promising antineoplastic and cardioprotective agent.

### 5.2. Dexrazoxane

Dexrazoxane is the first approved treatment by FDA specific to anthracycline-induced cardiotoxicity. It is a water-soluble analog of the iron chelator ethylenediaminetetraacetic acid (EDTA), and is capable of decreasing anthracycline-induced ROS generation through iron sequestration and iron replacement from anthracycline binding sites. Furthermore, increasing studies have demonstrated that the suppression and reduction of topoisomerase IIβ caused by dexrazoxane and its iron chelating metabolite ADR-925 also plays an important role in its cardioprotective effect (108). Besides, it's reported that dexrazoxane can prevent myocardial cell apoptosis and necrosis through suppressing the MAPK/NF-kB pathways, thus decreasing doxorubicin-mediated inflammation (109).

Recently, Noel et al. reported that mouse models receiving dexrazoxane during doxorubicin therapy showed dramatically reduced myocardial edema, improved GLS, and decreased myocardial deformation compared with no dexrazoxane through continuous monitoring of cardiac function by MRI (110). The result confirmed the cardiac protective effect of dexrazoxane. Clinical trials also validated the effect of dexrazoxane in lowering the incidence of HF (11% vs. 1%) and cardiac events (39% vs. 13%) in advanced or metastatic breast cancer (111).

Notably, early studies have shown that dexrazoxane may elevate the risk of secondary malignant tumors and reduce the efficacy of concomitant anti-tumor drugs (112). Nevertheless, increasing studies demonstrated the cardiac protection effect of dexrazoxane with no interference in the risk for secondary malignancies or the action of anticancer drugs. For instance, Asselin et al. demonstrated that the use of dexrazoxane rescued the LV wall thickness and the LVEF reduction measured by echocardiogram in children and adolescents suffering from acute lymphocytic leukemia or non-Hodgkin lymphoma undergoing anthracycline treatment, with no compromised antitumor efficacy or increased risk of second malignancies (113). Notably, the FDA approved application of dexrazoxane is confined to cardioprotection in advanced or metastatic breast cancer with ongoing anthracycline administration upon an overall dose of over 300 mg/m^2^ of doxorubicin (114).

Encouragingly, Chow et al. recently provided longer-term data of dexrazoxane on HF and cardiomyopathy in survivors of childhood cancer treated with doxorubicin. Results showed that amid young childhood cancer survivors, dexrazoxane was proven a cardioprotective role approximately 20 years later than first anthracycline treatment (NCT01790152) (115). Macedo et al. performed a meta-analysis including 7 randomized trials and 2 retrospective studies with an overall of 2,177 patients who were treated with anthracyclines. Dexrazoxane reduced the risk of clinical HF and cardiac events in patients with breast cancer undergoing anthracycline chemotherapy with or without trastuzumab and did not significantly impact cancer outcomes such as oncological response, overall survival, and progression-free survival. However, the quality of available evidence is low, and further randomized trials are warranted before the systematic implementation of this therapy in clinical practice (114).

### 5.3. Statins

Statins are drugs that lower cholesterol and prevent heart attacks and strokes. Given their cardiac protective effect, statins have been studied as a primary prevention of anthracycline-induced cardiotoxicity. Huelsenbeck et al. suggested that, in mouse models, lovastatin reduced doxorubicin-caused acute heart injuries, which was verified by decreased mRNA levels of the pro-fibrotic cytokine connective tissue growth factor (CTGF). And lovastatin prevented doxorubicin-induced subacute cardiac injuries as well, which was verified by declined mRNA levels of CTGF and atrial natriuretic peptide. In addition, increased serum cTnI level and enhanced cardiac fibrosis after doxorubicin administration were also alleviated through lovastatin (116). Kim et al. found that compared with carvedilol, rats pre-treated with rosuvastatin were observed less LV fibrosis, LVEF decline, and cTn increase upon four-week doxorubicin withdrawal (117).

Apart from these pre-clinical studies, some clinical trials have investigated the role of statins in primary prevention of anthracycline-induced cardiotoxicity in various cancers. In a randomized controlled trial of 40 patients with various cancers, prophylactic use of statin for 6 months after anthracycline therapy exerted a protective role on myocardium, with the placebo group suffering from evidently decreased LVEF (from 62.9% to 55.0%, *p* < 0.0001), but no alteration in LVEF was observed in the atorvastatin arm (from 61.3% to 62.6%, *p* = 0.144) (118). Likewise, in a prospective study involving 51 patients with different tumors, patients with no statin administration exhibited a declined LVEF (from 57.5% to 52.4%, *p* = 0.0003), however no remarkable alteration in LVEF was found in patients with statin treatment (from 56.6% to 54.1%, *p* = 0.15) after 6 months on CMR (119). Recently, Abdel-Qadir et al. studied the relationship between statin treatment and hospitalization or emergency department visits (hospital presentations) for HF after receiving anthracyclines for early breast cancer. Their work indicated that women treated with statin showed a lower rate of HF hospital presentations upon receiving anthracyclines (120).

Although previous studies provided some evidence supporting statins, current research is not adequate to recommend statin as prevention of anthracycline-caused cardiotoxicity. Therefore, further studies are demanded to determine whether there is a clear role of statins in preventing anthracycline-induced cardiotoxicity.

### 5.4. Angiotensin converting enzyme inhibitors (ACEIs) and angiotensin receptor blockers (ARB)

Clinical trial data for ACEIs and ARB treatment in preventing anthracycline-induced cardiotoxicity has been mixed. Wittayanukorn et al. (121) employed the Surveillance, Epidemiology and End-Results-Medicare-linked (SEER) database to choose 6,542 women with breast cancer undergoing anthracyclines or trastuzumab administration. Patients receiving ACEIs before or within 12 months upon the beginning of anthracyclines/trastuzumab (*n* = 508) were compared to those who never received ACEIs treatment (*n* = 6,034). Patients exposed to ACEIs showed 23% lower rate of suffering from cardiotoxicity and 21% lower rate of all-cause death, though these patients appeared to be less healthy with more preexisting cardiovascular risk factors and more comorbidities. Starting ACEIs ≤6 months after the beginning of anthracyclines or trastuzumab and having exposed duration ≥6 months were related to the reduced risk of cardiotoxicity and death as well.

Cardinale et al. performed a large investigation on enalapril in preventing anthracycline-induced cardiotoxicity—the International Cardio Oncology Society-one trial (ICOS-ONE), testifying two strategies: enalapril in all patients starting prior to anthracycline therapies (prevention group) and enalapril started merely in patients with an elevation in cTn during or after anthracycline therapies (cTn-triggered group) (122). Enalapril was administered for 1 year. Results showed that low overall doses of anthracyclines in patients with low cardiovascular risk could elevate cTn, with no difference between the two strategies of enalapril administration. Given the effect of enalapril on protecting against LV dysfunction, a cTn-triggered strategy is probably more convenient. On the other hand, considering that cTn level may rise even after low dose of anthracyclines and is not closely related to the incidence of cardiotoxicity, enalapril exposure independent from cTn level may be more suitable in clinical practice.

A large trial assessing angiotensin antagonists in preventing anthracycline-induced cardiotoxicity called PRADA trial (123) was carried out on 120 breast cancer patients undergoing anthracycline administration. Gulati et al. detected and compared the levels of cardiac cTnI, cTnT, BNP, NT-proBNP, C-reactive protein (CRP) and galectin-3 between control and candesartan arms. The average levels of these biomarkers elevated until accomplishment of anthracyclines, however, merely cTn and CRP levels were proportional to the dose of anthracyclines. Moreover, the trial demonstrated that combined treatment with candesartan avoided LVEF reduction caused by anthracyclines—in the control arm, the reduction was 2.6% and 0.8% in the experimental arm. No difference in right ventricular ejection fraction was observed, as identified through MRI, neither in the LV peak systolic global longitudinal strain via 2D speckle tracking imaging. Notably, the degree of LVEF reduction was lower than what the researchers originally expected; therefore, the potence of the research was diluted to determine the differences between arms.

In conclusions, most of studies (as shown in Table 2) demonstrated the protective role of RAS inhibitors, but requiring to be further confirmed in larger randomized trials with great emphasis on the clinically relevant end points such as mortality and occurrence of HF. Longer follow-up are essential to accurately determine the degree of cardiotoxicity prevention realized by ACEIs/ARB. And it is required to further characterize of high-risk patients and conduct studies in these populations.

**Table 2:** Clinical trials evaluating ACEIs/ARB for preventing anthracycline-induced cardiotoxicity.

Therapies	Cancer	Trial design	Follow up	Results	Ref.
Placebo vs. candesartan vs. metoprolol vs. candesartan + metoprolol	Breast cancer	2 × 2 factorial, randomized, placebo-controlled, double-blind trial (*n* = 130)	3–5 months	Overall decline in LVEF: 2.6 percentage points in the placebo group, 0.8 in the candesartan group, no effect in the metoprolol group	Gulati et al. (123)
Enalapril started before chemotherapy vs. enalapril started merely in patients with elevated cTn	Various cancers, 76% with breast cancer	Multicenter randomized, controlled trial (*n* = 273)	12 months	Incidence of troponin elevation was 23% in the prevention and 26% in the troponin-triggered group	Cardinale et al. (122)
Enalapril vs. placebo	Breast cancer; Hodgkin's lymphoma; Wilms tumor; Lung cancer; Bone sarcoma	Randomized, single-blind, and placebo-controlled (*n* = 69)	6 months	LVEF was not affectd in the enalapril arm, but remarkably reduced in placebo arm. Besides, LV end systolic volume and left atrial diameter were remarkably raised in contrast to the placebo arm	Janbabai et al. (124)
Enalapril vs. placebo	Leukemia (*n* = 41) and lymphoma (*n* = 43)	Randomized, double-blind, placebo-controlled trial (*n* = 84)	6 months	LVEF reduced in both arms after 6 months, more so in placebo arm (62.25 ± 5.49 vs. 56.15 ± 4.79); cardiac biomarkers rose more in placebo arm after 6 months, especially for proBNP and cTnI while not remarkable for CK-MB	Gupta et al. (125)
Ramipril and/or bisoprolol vs. control (retrospective group without primary cardioprotection)	Non-Hodgkin lymphoma (NHL)	Controlled trial [experimental group (*n* = 35) and control group (*n* = 62)]	18 months while retrospective group followed up for 36 months	NHL patients with a primary cardioprotection strategy did not experience cardiovascular deaths compared with the retrospective control group where cardiovascular mortality was 14.5% at 3 years. Primary cardioprotection also decreased the frequency of new cardiotoxicity-related clinical symptoms (2.8% vs. 24.1%) and prevented the occurrence of cardiac systolic dysfunction (0% vs. 8.5%)	Długosz-Danecka et al. (126)

### 5.5. Beta-blockers

Beta blockers have a well-established effect on guideline-directed treatment of HF given their neurohormonal action such as lowering heart rate and alleviating arrhythmias. Besides, certain beta blockers like carvedilol and nebivolol, show prominent antioxidant properties which deplete ROS and protect against mitochondrial disorders, indicating a robust potential in avoiding anthracycline-induced cardiotoxicity (127). A clinical trial reported that the addition of nebivolol for patients with breast cancer undergoing anthracyclines led to improved LVEF, reduced LV end-systolic and end-diastolic diameters, and declined BNP levels after 6 months compared with the control arm (128).

Notably, remarkable heterogeneity exists between studies, most of which are conducted on small samples with different methodology, various definitions of cardiotoxicity and absence of long-term monitoring of clinical effect, so the statistical power to determine the action of beta blockers in protecting against anthracycline-mediated cardiomtoxicity is inadequate. For instance, a study by Nabati and coworkers studied the effect of carvedilol on protecting against doxorubicin-caused cardiotoxicity. Ninety-one women with recently diagnosed breast cancer receiving doxorubicin treatment were randomized into arms giving either carvedilol or placebo. No alteration in LVEF was found in the carvedilol arm, while LVEF was remarkably declined in the placebo arm. Furthermore, echocardiography indicated that both LV end systolic volume and LA diameter were remarkably raised in contrast to the placebo arm (129).

Nevertheless, a large prospective randomized clinical trial called CECCY study evaluated the effect of carvedilol on protecting against anthracycline-induced cardiotoxicity, and testified that in contrast to placebo, carvedilol attenuated the progression of anthracycline-mediated cardiomyocyte injuries, accompanied by decreased troponin levels and alleviated diastolic dysfunction (130). In another study, Huang et al. suggested that the prophylactic application of carvedilol had no influence in the early asymptomatic LVEF reduction while probably could lower the incidence of evident cardiotoxicity and protect against ventricular remodeling (131). Interestingly, in contrast to third generation beta blockers like nebivolol and carvedilol, metoprolol hasn't demonstrated prominent antioxidant effects. In the beta blocker monotherapy arm of the PRADA study, metoprolol succinate exerted no influence in decrease of LVEF or GLS in short or long-term follow up (132). Collectively, current studies (as shown in Table 3) suggest a prospective effect of beta blockers, especially carvedilol and nebivolol, but the small sample and short follow up of the most research confine the extensive use to all patients receiving anthracyclines.

**Table 3:** Clinical trials evaluating beta-blockers for the preventing anthracycline-induced cardiotoxicity.

Therapies	Cancer	Trial design	Follow up	Results	Ref.
Carvedilol or placebo	Breast cancer	Prospective, randomized, double-blind, placebo-controlled (*n* = 200)	6 months	No differences in changes of LVEF or B-type natriuretic peptide were noted between groups. A significant difference existed between groups in troponin I levels, with lower levels in the carvedilol group. Additionally, a lower incidence of diastolic dysfunction was noted in the carvedilol group	Avila et al. (130)
Placebo vs. candesartan vs. metoprolol vs. candesartan + metoprolol	Breast cancer	2 × 2 factorial, randomized, placebo-controlled, double-blind trial (*n* = 130)	3–5 months	No significant effect on change in LVEF with metoprolol vs. Placebo	Gulati et al. (123)
Carvedilol vs. placebo	Breast cancer	Double-blind randomized trial (*n* = 70)	1 week	No significant reduction in strain and strain-rate parameters upon intervention, but an obvious decrease in these parameters was observed in the placebo arm	Tashakori Beheshti et al. (133)
Carvedilol vs. placebo	Breast cancer	Randomized, single-blind, placebo-controlled (*n* = 91)	6 months	LVEF did not alter in the carvedilol arm (from 58.7% to 57.4%), but remarkably decreased in the placebo arm (from 61.1% to 51.6%)	Nabati et al. (129)
Carvedilol [6.25 mg/day (*n* = 41), 12.5 mg/day (*n* = 38) or 25 mg/day (*n* = 37)] vs. placebo (*n* = 38)	Various cancers	Prospective, randomized, double-blind (*n* = 154)	6 months	LVEF reduced from 62 ± 5% to 58 ± 7% at 6 months in the control arm, however no significant alterations occurred in the 3 carvedilol arms	Abuosa et al. (134)

Additionally, it has been proposed that combined beta blocker and ACEIs treatment may exhibit an improved synergistic action compared with monotherapy, nevertheless, the outcomes are contradictory among studies. In the PRADA study, no remarkable alterations in LVEF, GLS, or LVESD were observed in breast cancer patients receiving concomitant treatment with candesartan and metoprolol compared with the control arm both in short and long-term follow up (132). However, the OVERCOME study observed a benefit of concomitant treatment with enalapril and carvedilol in acute leukemia patients without remarkable alterations in LVEF after 6 months, as compared to 3% LVEF decline in the control arm (135). Altogether, larger studies are demanded to confirm the clinical relevance of this strategy for preventing anthracycline-induced cardiotoxicity through combined ACEIs and beta blocker treatment.

## 6. Conclusions and perspectives

The incidence of cardiotoxicity related to anthracycline chemotherapy is anticipated to increase steadily in the near future, considering an increasing number of cancer survivors and the gradual ageing of the population which lead to a growing number of patients aged 65 years or above who have elevated cardiovascular risk and probably suffer from another cardiac damage caused by anthracycline-mediated cardiotoxicity. Therefore, early detection and long-term follow up of anthracycline-induced cardiotoxicity via monitor means, particularly the advanced multimodality imaging modalities reviewed in this article may enable intervention at an earlier stage, which is vital to improve patient's prognosis and reduction in death risk. Prevention measures have not been fully verified. Research has found that ergothioneine may be a viable co-therapy to alleviate the cardiotoxic effects of anthracyclines in the treatment of cancers (136). Dexrazoxane has showed its effect on the attenuation of anthracycline-caused cardiotoxicity, but its safety and influence in cancer-associated outcomes are still debatable. A limited number of clinical trials carried out with statins showed prospective outcomes. Trials studying ACEIs and ARB exhibit supportive outcomes in protecting against LV function reduction, but they didn't mitigate the elevation of cTn, indicating that RAS is not participated in the direct cardiotoxicity induced by anthracyclines but exerts an effect on the myocardial remodeling which occurs after anthracycline treatment. In contrast, betablockers were capable of mitigating the elevation of troponin in some trials, however they showed inconsistent outcomes in preventing LVEF reduction. Therefore, multicenter large clinical trials on the aforementioned drug are vital to achieve better understanding and prevention of anthracycline-induced cardiotoxicity. Further advance of oncological cardiology needs more emphasis on high-risk groups (as show in Table 4), heart function monitoring, and preventive and therapeutic measures. Moreover, the mechanism of anthracycline-induced cardiotoxicity should be explored further to identify methods for better preventing anthracycline-induced cardiotoxicity. We should upgrade existing anthracycline, or develop more efficient and low toxicity anthracycline.

**Table 4:** Risk factors for developing anthracycline-induced cardiotoxicity.

Patient-related risk factors	• Preexisting cardiovascular risks, for instance, arterial hypertension, diabetes, smoking, renal failure, baseline left ventricular dysfunction, valvular heart disease, coronary disease, etc.
	• Female gender
	• Age >65 years or <18 years
	• African-American ancestry
	• Genetic factors
Therapy-related risk factors	• Cumulative dose of anthracyclines
	• Infusion regimen (For adults, continuous infusion is usually more tolerant compared with bolus dosing)
	• Concomitant use of other anticancer treatments like radiotherapy or trastuzumab
